# Supplementary material for: High Expression of Casein Kinase 2 Alpha Is Responsible for Enhanced Phosphorylation of DNA Mismatch Repair Protein MLH1 and Increased Tumor Mutation Rates in Colorectal Cancer
Source: Cancers (Basel). 2022 Mar 18;14(6):1553. doi: 10.3390/cancers14061553 (PMC8946085; doi:10.3390/cancers14061553)
Supplement: Supplementary file 1 [file cancers-14-01553-s001.zip › Figures S3-6_Western blots corresponding to Figure 4A-D.pdf]

Figure S3

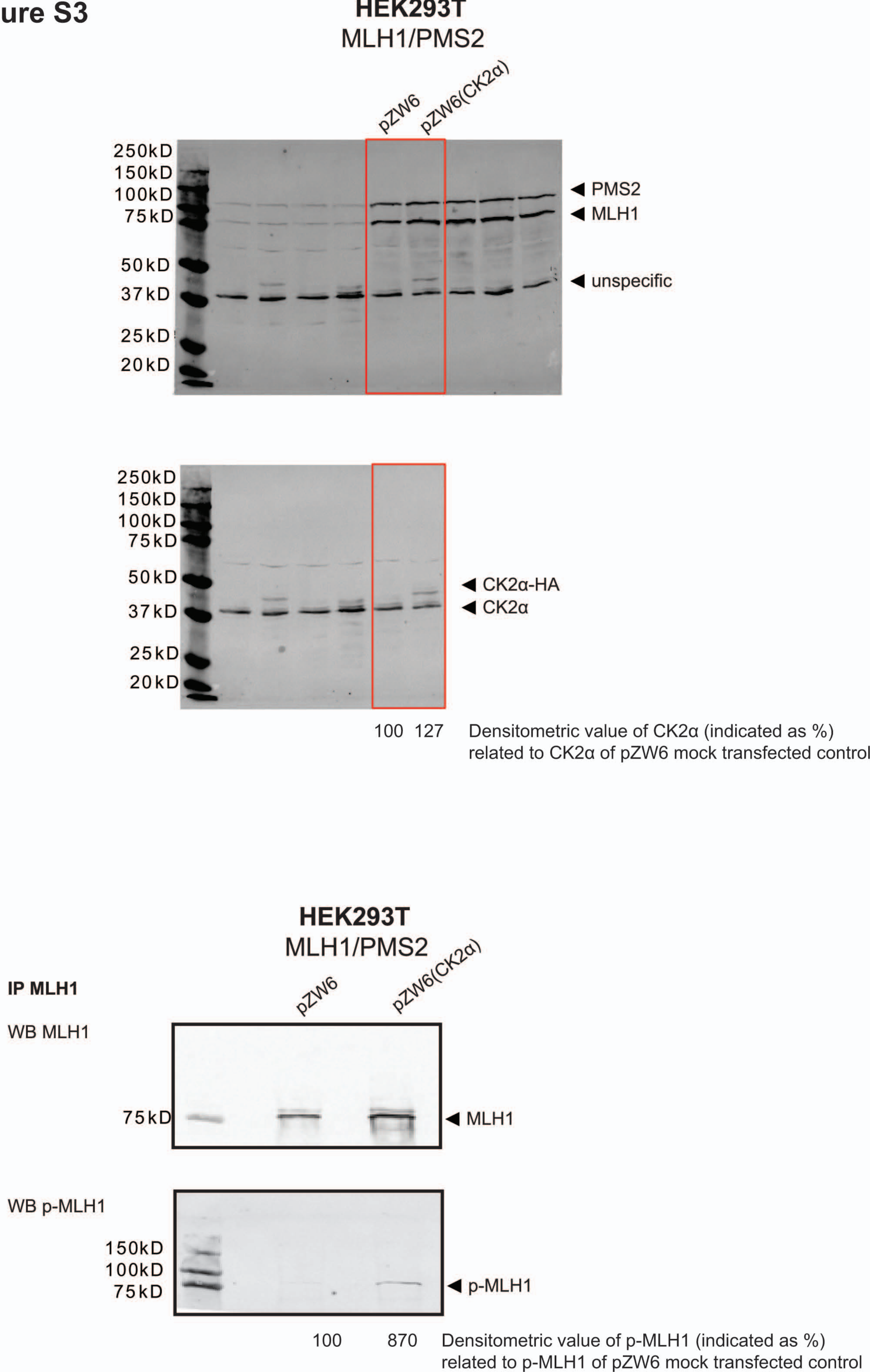
HEK293T
MLH1/PMS2
pZW6
pZW6(CK2α)
250kD
150kD
100kD
75kD
50kD
37kD
25kD
20kD
PMS2
MLH1
unspecific
250kD
150kD
100kD
75kD
50kD
37kD
25kD
20kD
CK2α-HA
CK2α
100 127
Densitometric value of CK2α (indicated as %)
related to CK2α of pZW6 mock transfected control
HEK293T
MLH1/PMS2
IP MLH1
pZW6
pZW6(CK2α)
WB MLH1
75kD
MLH1
WB p-MLH1
150kD
100kD
75kD
p-MLH1
100
870
Densitometric value of p-MLH1 (indicated as %)
related to p-MLH1 of pZW6 mock transfected control

Figure S4

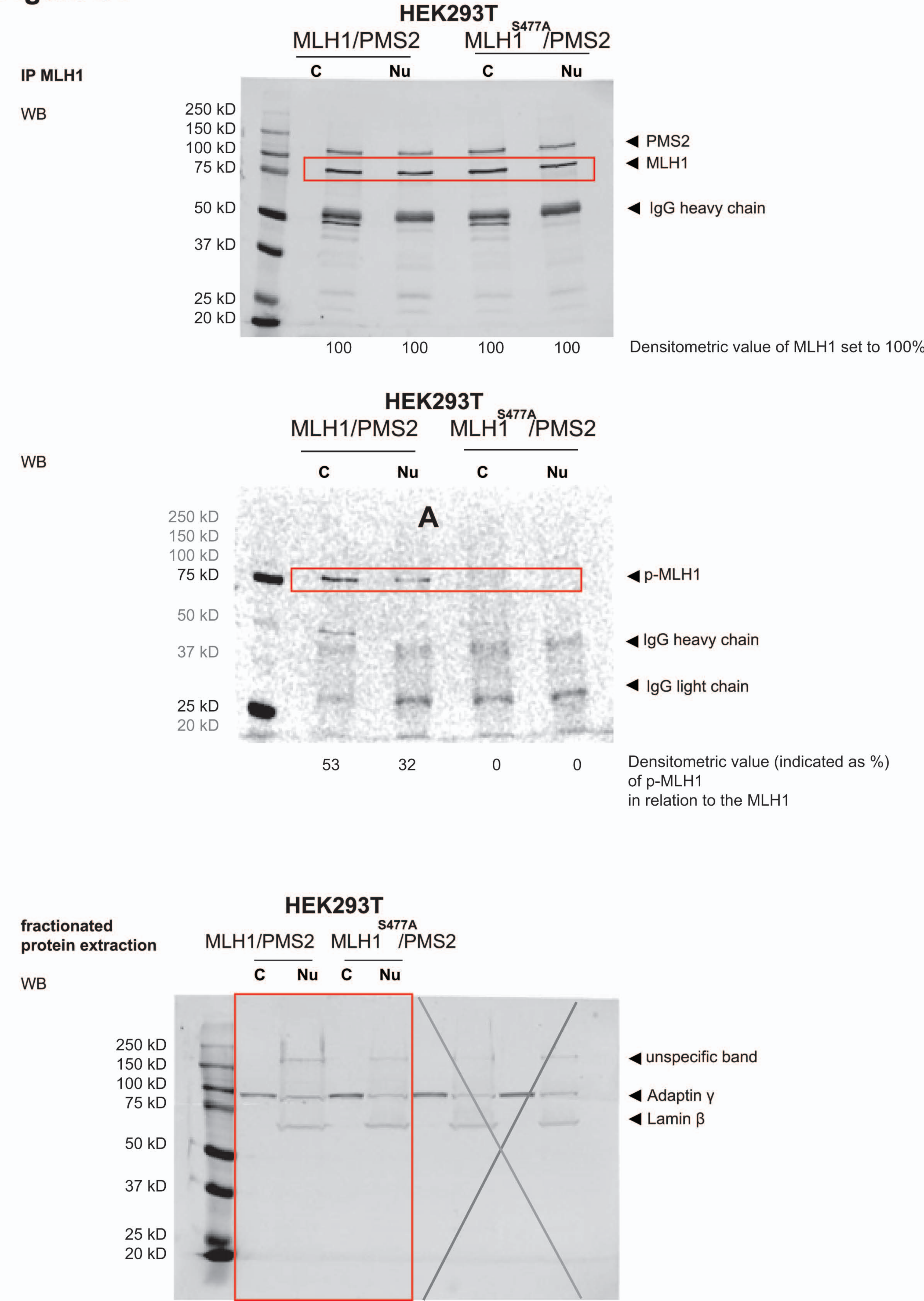

HEK293T
MLH1/PMS2
MLH1 S477A /PMS2
IP MLH1
WB
C
Nu
C
Nu
250 kD
150 kD
100 kD
75 kD
50 kD
37 kD
25 kD
20 kD
PMS2
MLH1
IgG heavy chain
100
100
100
100
Densitometric value of MLH1 set to 100%
HEK293T
MLH1/PMS2
MLH1 S477A /PMS2
WB
C
Nu
C
Nu
A
250 kD
150 kD
100 kD
75 kD
50 kD
37 kD
25 kD
20 kD
p-MLH1
IgG heavy chain
IgG light chain
53
32
0
0
Densitometric value (indicated as %)
of p-MLH1
in relation to the MLH1
HEK293T
fractionated
protein extraction
MLH1/PMS2
MLH1 S477A /PMS2
WB
C
Nu
C
Nu
250 kD
150 kD
100 kD
75 kD
50 kD
37 kD
25 kD
20 kD
unspecific band
Adaptin γ
Lamin β

Figure S5

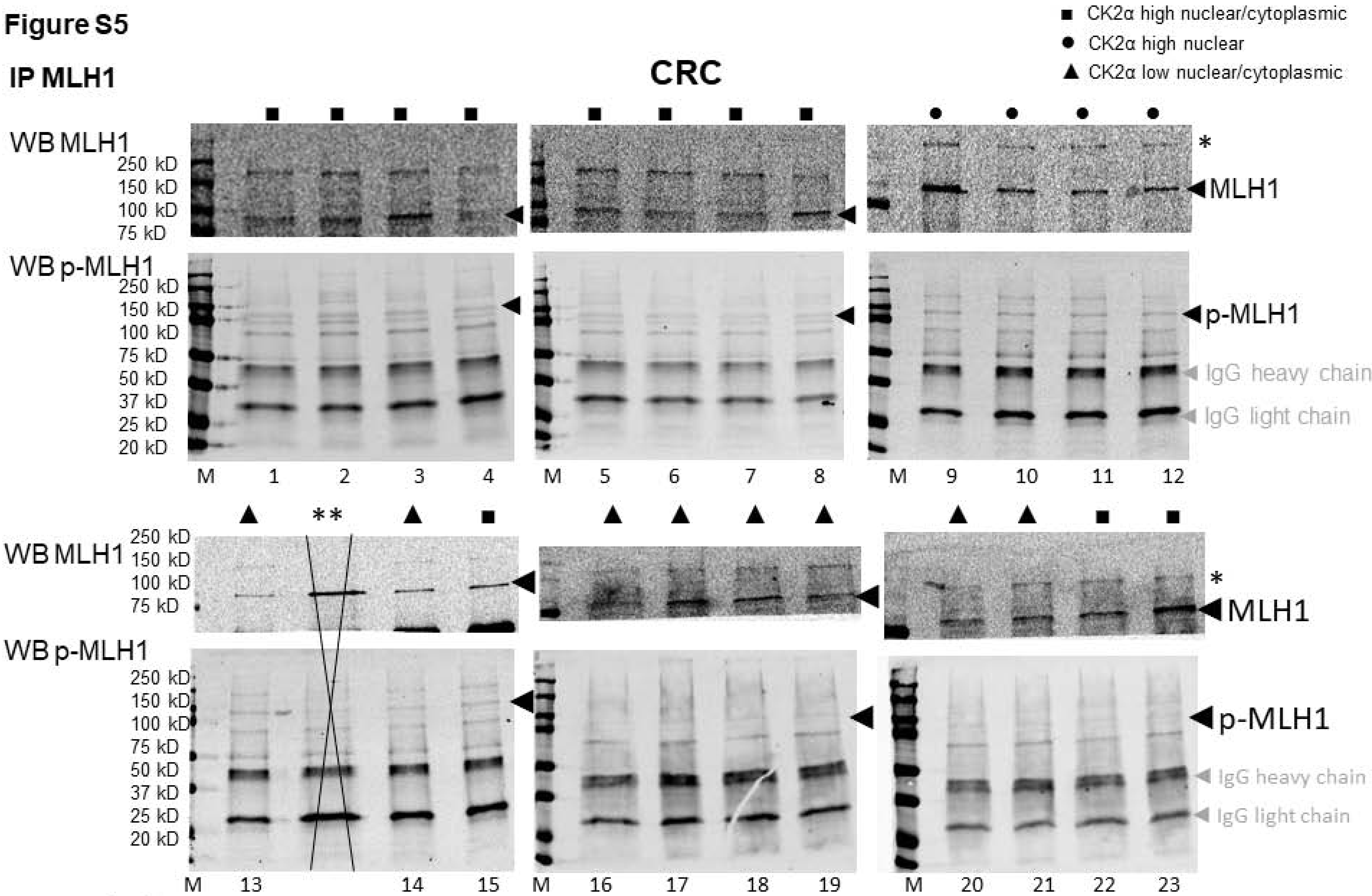

* unspecific binding

** sample not included in the evaluation

M: Marker; 1: patient 93; 2: patient 103; 3:patient 85; 4: patient 43; 5: patient 49; 6: patient 50; 7:patient 125; 8: patient 132; 9: patient 89; 10: patient 44; 11: patient 98; 12: patient 116; 13: patient 59; 14: patient 148; 15: patient 26; 16: patient 82; 17: patient 23; 18: patient 10; 19: patient 135; 20: patient 52; 21: patient 162; 22: patient 139; 23: patient 133; (see supplementary table 1).

Figure S6

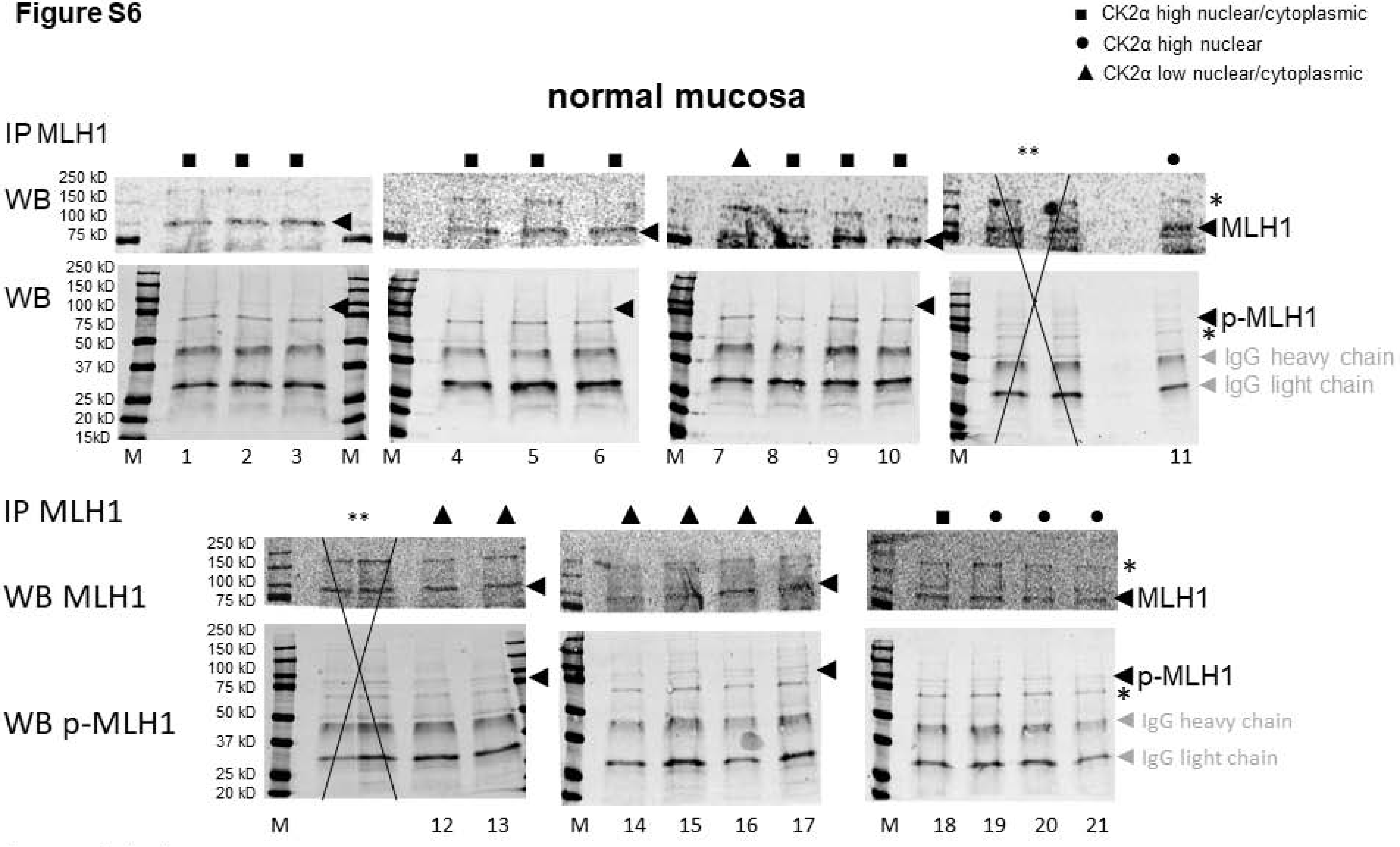

* unspecific binding

** sample not included in the evaluation

M: Marker; 1: patient 93; 2: patient 103; 3: patient 85; 4: patient 125; 5: patient 50; 6: patient 43; 7: patient 23; 8: patient 139; 9: patient 133; 10: patient 132; 11: patient 98; 12: patient 82; 13: patient 52; 14: patient 10; 15: patient 135; 16: patient 59; 17: patient 148; 18: patient 26; 19: patient 116; 20: patient 44 ; 21: patient 89; (see supplementary table 1).
