# Supplementary material for: High Expression of Casein Kinase 2 Alpha Is Responsible for Enhanced Phosphorylation of DNA Mismatch Repair Protein MLH1 and Increased Tumor Mutation Rates in Colorectal Cancer
Source: Cancers (Basel). 2022 Mar 18;14(6):1553. doi: 10.3390/cancers14061553 (PMC8946085; doi:10.3390/cancers14061553)
Supplement: Supplementary file 1 [file cancers-14-01553-s001.zip › supplementary.pdf]

Table S1.

Clinicopathological characteristics of 165 Patients with colorectal carcinomas evaluated for MLH1 and CK2α expression

Patient-ID	Gender[1]	Age at diagnosis	Year of diagnosis and surgery	Localisation relative to the splenic flexure	Histology	T-Stage	Metastases[2]	UICC-Stage	MLH1	Mucosa (CKα Intensity)	Tumor (CK2α Intensity)	Change %	Compared to Mucosa
1	F	34	2011	Distal	Mucin-producing Adenocarcinoma	T1	M0	I	+	1.17126	1.24649	6.42299746	=
2	F	62	2014	Proximal	Adenocarcinoma	T2	M0	I	+	1.1171	1.67448	49.8952645	>
3	M	77	2012	Proximal	Adenocarcinoma	T1	M0	I	+	1.21708	1.23166	1.19794919	=
4	M	84	2015	Distal	Adenocarcinoma	T3	M0	IIA	+	1.58197	1.60601	1.51962427	=
5	M	75	2014	Proximal	Adenocarcinoma	T4b	M0	IIC	+	1.19294	1.24659333	4.49757188	=
6	F	74	2014	Distal	Adenocarcinoma	T3	M0	IIIB	+	1.32043	1.57811	19.514855	>
7	F	69	2015	Proximal	Adenocarcinoma	T3	M0	IIIB	+	1.2856	1.67579333	30.3510683	nuclear
8	M	79	2012		Adenocarcinoma	T2	M1a (HEP)	IVA	+	1.24398	1.61259333	29.6317733	>
10	M	77	2014	Proximal	Adenocarcinoma	T1	M0	I	+	1.15656	1.16927	1.09894861	=
11	F	49	2014	Distal	Adenocarcinoma	T1	M0	I	+	1.1747	1.26819	7.95862773	=
12	F	69	2011	Proximal	Adenocarcinoma	T1a	M0	I	+	1.27696	1.26750667	-0.7402999	=
13	M	73	2013	Distal	Adenocarcinoma	T1	M0	I	+	1.25452	1.53653	22.4795141	>
14	M	88	2013	Proximal	Adenocarcinoma	T2	M0	I	+	1.22632	1.28052	4.41972731	=
15	F	58	2013	Distal	Adenocarcinoma	T2	M0	I	+	1.23136	1.33752	8.62136175	=
16	F	57	2013	Distal	Adenocarcinoma	T1	M0	I	+	1.20786	1.28052	6.01559783	=
17	F	74	2015	Proximal	Adenocarcinoma	T2	M0	I	+	1.29815	1.86618	43.7568848	>
18	M	75	2012	Distal	Adenocarcinoma	T2	M0	I	+	1.19904	1.60978	34.2557379	>
19	M	70	2012	Proximal	Adenocarcinoma	T2	M0	I	+	1.20989	1.34314	11.0133979	>
20	F	65	2013	Proximal	Adenocarcinoma	T1	M0	I	+	1.18025	1.49458	26.6324931	>
21	M	60	2015	Distal	Adenocarcinoma	T2	M0	I	+	1.53812	2.03883334	32.553594	>
22	M	52	2014	Distal	Adenocarcinoma	T2	M0	I	+	1.17725	1.15087	-2.2408155	=
23	F	61	2014	Proximal	Adenocarcinoma	T2	M0	I	+	1.16185	1.16409	0.19279597	=
24	M	65	2013	Proximal	Adenocarcinoma	T2	M0	I	+	1.17882	1.54734	31.2617702	>

25	M	78	2014	Proximal	Adenocarcinoma	T2	M0	I	+	1.28341	1.72041	34.0499139	>
26	M	72	2015	Proximal	Adenocarcinoma	T2	M0	I	+	1.42492	1.60871	12.898268	>
27	M	76	2014	Distal	Adenocarcinoma	T2	M0	I	+	1.59102	1.77016	11.2594436	>
28	M	84	2012	Proximal	Adenocarcinoma	T2	M0	I	+	1.21594	1.3186	8.4428508	=
29	M	60	2011	Proximal	Adenocarcinoma	T1	M0	I	+	1.43408	1.77889	24.0439864	>
30	F	59	2012	Distal	Adenocarcinoma	T2	M0	I	+	1.26306	1.22832	-2.7504632	=
32	M	91	2014	Distal	Adenocarcinoma	T2	M0	I	+	1.2301	1.46936	19.4504512	>
33	M	60	2013	Proximal	Adenocarcinoma	T3	M0	IIA	+	1.69152	1.75247	3.60326807	=
34	M	76	2013	Distal	Adenocarcinoma	T3	M0	IIA	+	1.10714	1.4711	32.8738913	>
35	M	78	2015	Distal	Adenocarcinoma	T3	M0	IIA	+	1.22463	1.27628	4.21760042	=
36	M	78	2016	Proximal	Adenocarcinoma	T3	M0	IIA	+	1.53195	1.30507333	-14.809665	nuclear
38	M	60	2012	Distal	Adenocarcinoma	T3	M0	IIA	+	1.16464	1.25744	7.96812749	=
39	F	85	2013	Proximal	Adenocarcinoma	T3	M0	IIA	+	1.18476	1.67889	41.7071812	>
41	M	87	2014	Proximal	Adenocarcinoma	T3	M0	IIA	+	1.17648	1.6111	36.9424045	>
42	F	70	2015	Proximal	Adenocarcinoma	T3	M0	IIA	+	1.78906	2.21338666	23.7178552	>
43	F	74	2012	Distal	Adenocarcinoma	T3	M0	IIA	+	1.9104	2.60082	36.1400754	>
44	M	74	2011	Proximal	Adenocarcinoma	T3	M0	IIA	+	0	1.83529		nuclear
45	M	76	2013	Proximal	Adenocarcinoma	T3	M0	IIA	+	1.19438	1.26372	5.80552253	=
46	F	63	2016	Proximal	Adenocarcinoma	T3	M0	IIA	+	1.33194	1.44055	8.15427121	nuclear
47	M	51	2016	Proximal	Adenocarcinoma	T3	M0	IIA	+	1.19828	1.31621	9.8416063	>
48	M	52	2014	Distal	Adenocarcinoma	T3	M0	IIA	+	1.85072	1.89702	1.02501729	=
49	F	73	2012	Distal	Adenocarcinoma	T3	M0	IIA	+	1.85635	2.53316666	36.4595394	>
50	F	73	2012	Proximal	Adenocarcinoma	T3	M0	IIA	+	2.2243	2.53765	14.0875781	>
51	M	83	2014	Distal	Adenocarcinoma	T3	M0	IIA	+	1.37046	1.5807	15.3408345	>
52	M	69	2013	Proximal	Adenocarcinoma	T3	M0	IIA	+	1.22305	1.14596667	-6.3025497	=
53	M	53	2012	Proximal	Adenocarcinoma	T3	M0	IIA	+	1.39278	2.08381	49.6151582	>
54	F	75	2015	Proximal	Mucinous adenocarcinoma	T3	M0	IIA	+	1.24587	1.90461333	52.874163	>
55	M	87	2012	Proximal	Adenocarcinoma	T3	M0	IIA	+	1.1878	1.19259	0.40326654	=
56	F	83	2013	Proximal	Adenocarcinoma	T3	M0	IIA	+	1.21637	1.28531	5.66768335	=

57	M	58	2011	Proximal	Adenocarcinoma	T3	M0	IIA	+	1.23962	1.51942	22.5714332	nuclear
58	M	81	2013	Distal	Adenocarcinoma	T3	M0	IIA	+	1.14288	1.59426	39.4949601	>
59	F	77	2012	Proximal	Mucinous adenocarcinoma	T3	M0	IIA	+	1.2215	1.26612	3.6528858	=
60	M	72	2012	Distal	Adenocarcinoma	T3	M0	IIA	+	1.15784	1.32974	14.8466109	>
62	F	62	2012	Proximal	Adenocarcinoma	T3	M1 (PUL)	IIA	+	1.16268	1.30233	12.0110435	>
63	M	80	2012	Distal	Adenocarcinoma	T4a	M0	IIB	+	1.18917	1.1638	-2.1334208	=
65	M	78	2013	Proximal	Adenocarcinoma	T4b	M0	IIC	+	1.38556	1.90866	37.753688	>
66	M	81	2013	Distal	Adenocarcinoma	T4b	M0	IIC	+	1.16827	1.24809	6.83232472	=
67	M	75	2013	Distal	Mucinous adenocarcinoma	T4b	M0	IIC	+	1.17509	1.29089	9.85456433	>
68	M	65	2016	Distal	Adenocarcinoma	T4b	M0	IIC	+	1.59946	2.37050666	48.206686	>
72	M	51	2015	Distal	Adenocarcinoma	T1	M0	IIIA	+	1.25233	1.45175	15.9239178	>
73	M	79	2014	Distal	Adenocarcinoma	T2	M0	IIIA	+	1.30643	2.20211334	68.5596121	>
74	F	38	2016	Distal	Adenocarcinoma	T2	M0	IIIA	+	1.57366	1.72437	9.57703697	>
75	F	87	2010	Proximal	Adenocarcinoma	T2	M0	IIIA	+	1.72505	1.75683333	1.84245871	=
76	F	75	2015	Distal	Adenocarcinoma	T2	M0	IIIA	+	1.77193	2.56805	44.9295401	>
78	F	69	2015	Distal	Adenocarcinoma	T3	M0	IIIB	+	1.45165	1.73616	19.5990769	nuclear
79	F	86	2014	Distal	Adenocarcinoma	T3	M0	IIIB	+	1.43986	1.60582	11.5261206	>
80	F	85	2013	Proximal	Adenocarcinoma	T3	M0	IIIB	+	1.22317	1.32724	8.50822044	=
81	M	63	2013	Proximal	Adenocarcinoma	T3	M0	IIIB	+	1.23482	1.33844	8.39150645	=
82	F	68	2016	Distal	Adenocarcinoma	T3	M0	IIIB	+	1.3656	1.45036	6.20679555	=
83	M	87	2014	Distal	Adenocarcinoma	T3	M0	IIIB	+	1.75389	2.20887	25.9411936	>
85	M	81	2014	Proximal	Adenocarcinoma	T3	M0	IIIB	+	1.57268	2.35295	49.6140346	>
86	F	83	2013	Proximal	Adenocarcinoma	T4a	M0	IIIB	+	1.41839	1.94472	37.1075656	>
87	F	74	2013	Distal	Adenocarcinoma	T3	M0	IIIB	+	1.23478	1.36114	10.2334019	>
88	M	65	2016		Adenocarcinoma	T3	M0	IIIB	+	1.38252	1.83649	32.8364147	>
89	F	87	2012		Adenocarcinoma	T3	M0	IIIB	+	1.38356	2.13061	53.9947671	nuclear
91	F	51	2013	Distal	Adenocarcinoma	T3	M0	IIIB	+	1.18697	1.23903	4.38595752	=
92	F	70	2013	Proximal	Adenocarcinoma	T3	M0	IIIB	+	1.24396	1.16747	-6.1489115	=
93	M	74	2016	Proximal	Adenocarcinoma	T3	M0	IIIB	+	1.43596	2.02692	41.1543497	>

94	M	81	2016	Distal	Adenocarcinoma	T3	M0	IIIB	+	1.55095	2.34952	51.4890873	>
95	F	77	2014	Distal	Adenocarcinoma	T3	M0	IIIB	+	1.36087	1.75141	28.6978183	nuclear
96	F	76	2011	Distal	Adenocarcinoma	T3	M0	IIIB	+	1.2862	2.02604	57.5213808	>
97	F	78	2015	Proximal	Adenocarcinoma	T3	M0	IIIB	+	1.31466	2.05092	56.0038337	>
98	F	91	2012	Proximal	Adenocarcinoma	T3	M0	IIIB	+	1.99426	2.51532	26.1279873	nuclear
99	F	67	2016	Proximal	Adenocarcinoma	T3	M0	IIIB	+	1.54585	2.30852666	49.3370418	>
101	M	78	2012	Proximal	Adenocarcinoma	T3	M0	IIIB	+	1.83908	2.32628	26.4915066	>
102	F	40	2012	Proximal	Adenocarcinoma	T4a	M1(HEP)	IIIB	+	1.1413	1.22910667	7.69356576	=
103	M	60	2012	Proximal	Adenocarcinoma	T4a	M0	IIIC	+	2.02064	2.55136666	26.2652754	>
104	F	68	2016	Proximal	Mucinous adenocarcinoma	T3	M0	IIIC	+	1.59261	1.82766	14.7587922	>
105	M	57	2014	Distal	Adenocarcinoma	T3	M0	IIIC	+	0	1.86904		nuclear
106	M	76	2011	Proximal	Adenocarcinoma	T3	M0	IIIC	+	1.17972	1.71838	45.6599871	>
107	F	71	2014	Distal	Adenocarcinoma	T3	M0	IIIC	+	1.20492	1.39847333	16.0635838	>
108	F	77	2014	Distal	Adenocarcinoma	T4b	M0	IIIC	+	1.60295	1.92804	20.2807324	>
112	F	41	2012	Proximal	Adenocarcinoma	T3	M1 (HEP)	IVA	+	1.21585	1.51256	24.4035037	nuclear
113	M	79	2013	Proximal	Adenocarcinoma	T3	M1a	IVA	+	1.39801	1.78919	27.9812019	>
114	M	81	2012	Distal	Adenocarcinoma	T4	M1a(HEP)	IVA	+	1.47887	1.97856667	33.7890867	>
115	F	77	2015	Distal	Mucin-producing Adenocarcinoma	T3	M1a (HEP)	IVA	+	1.73666	1.7	-2.1109486	=
116	M	75	2013	Proximal	Adenocarcinoma	T4b	M1a (HEP)	IVA	+	1.4503	1.67412	15.4326691	nuclear
117	M	46	2012	Proximal	Adenocarcinoma	T3	M1a (HEP)	IVA	+	1.19423	1.26796	6.17385261	=
119	F	76	2015	Proximal	Adenocarcinoma	T3	M1a (HEP)	IVA	+	1.20416	1.96718667	63.3658871	>
120	M	83	2012		Adenocarcinoma	T3	M1a (HEP)	IVA	+	1.34992	1.58081	17.1039765	>
121	M	64	2013	Distal	Adenocarcinoma	T3	M1a(OSS)	IVA	+	1.29076	1.71447	32.8263969	nuclear
122	F	83	2016	Proximal	Adenocarcinoma	T3	M1b	IVB	+	1.64802	2.77292	68.2576668	>
123	M	85	2012	Proximal	Adenocarcinoma	T3	M1b(HEP)	IVB	+	1.22001	1.41756667	16.1930366	nuclear
124	M	43	2013	Distal	Adenocarcinoma	T3	M1b(LYM)	IVB	+	1.11646	1.23396	10.5243358	>
125	M	86	2012	Proximal	Adenocarcinoma	T4	M1b (PER)	IVB	+	1.18927	2.16332	81.9031843	>
126	M	79	2012	Proximal	Adenocarcinoma	T3	M1b (PER)	IVB	+	1.347	1.52148	12.9532294	>
127	F	52	2013	Proximal	Adenocarcinoma	T4a	M1b (PER)	IVB	+	1.39447	2.29222	64.3792982	>

128	M	58	2014	Distal	Adenocarcinoma	T2	M0	I	+	1.58123	2.15428	36.2407746	>
129	M	80	2015	Distal	Adenocarcinoma	T2	M0	I	+	1.22875	1.70988	39.1560529	>
130	F	63	2016	Proximal	Adenocarcinoma	T2	M0	I	+	1.1992	1.65418	37.9402935	>
131	F	81	2016	Proximal	Adenocarcinoma	T2	M0	I	+	1.38294	2.23528	61.6324642	>
132	M	69	2016	Distal	Adenocarcinoma	T2	M0	I	+	2.02704	2.73835	35.0910688	>
133	F	61	2016	Distal	Adenocarcinoma	T2	M0	I	+	1.8443	2.89301	56.8622242	>
134	F	74	2014	Distal	Adenocarcinoma	T3	M0	II	+	1.59028	1.84258	15.8651307	>
135	M	57	2015	Proximal	Adenocarcinoma	T3	M0	II	+	1.62606	1.47918	-9.032877	=
137	F	53	2016	Proximal	Adenocarcinoma	T3	M0	II	+	1.49578	1.50866	0.8610892	nuclear
138	F	71	2016	Proximal	Adenocarcinoma	T3	M0	II	+	1.383	1.52588667	10.3316461	>
139	F	46	2013	Distal	Adenocarcinoma	T3	M1(HEP)	IV	+	1.24636	2.16909	74.033987	>
140	F	74	2014	Distal	Adenocarcinoma	T3	M1 (HEP)	IV	+	1.27477	1.88238	47.6642845	nuclear
141	F	54	2016	Proximal	Adenocarcinoma	T3	M1 (HEP)	IV	+	2.1494	2.7855	29.5943054	>
142	F	58	2016	Proximal	Adenocarcinoma	T3	M1 (HEP)	IV	+	1.19374	1.4861	24.4910952	nuclear
143	M	66	2013	Proximal	Adenocarcinoma	T3	M0	IIA	+	1.15985	1.32346	14.1061344	>
144	M	84	2014	Proximal	Adenocarcinoma	T3	M0	IIA	+	1.62922	2.0268	24.4030886	>
145	M	35	2016	Proximal	Adenocarcinoma	T3	M0	IIA	+	1.49085	1.5473	3.78643056	=
146	M	40	2013	Proximal	Adenocarcinoma	T3	M0	IIIB	+	1.14548	1.19885	4.65918218	nuclear
147	F	85	2011	Distal	Mucinous adenocarcinoma	T3	M0	IIIB	+	1.22202	1.21868	-0.2733179	=
148	F	71	2011	Proximal	Adenocarcinoma	T3	M1(PUL)	IIIB	+	1.34806	1.36452	1.2210139	=
149	F	73	2016	Proximal	Adenocarcinoma	T3	M0	IIIC	+	0	1.51851		nuclear
150	M	68	2015	Proximal	Neuroendocrine carcinoma	T4	M1	IVA	+	1.1851	1.32009	11.3905999	>
151	F	79	2016	Proximal	Adenocarcinoma	T4b	M1a	IVA	+	1.16472	1.15652	-0.7040319	=
152	F	65	2015	Proximal	Adenocarcinoma	T3	M1a(HEP)	IVA	+	1.53946	1.74139	13.1169371	>
153	M	56	2011		Adenocarcinoma	T3	M1a (HEP)	IVA	+	1.12769	1.16879	3.64461865	=
154	M	70	2015	Proximal	Adenocarcinoma	T4a	M1b	IVB	+	1.3545	1.37755333	1.7019811	=
155	M	62	2013	Distal	Adenocarcinoma	T4	M1b(LYM)	IVB	+	1.16523	1.43631	23.2640766	nuclear
156	F	52	2013	Distal	Adenocarcinoma	T4a	M1(HEP)	IV	+	1.1765	1.16282	-1.1627709	=
157	F	48	2013	Proximal	Adenocarcinoma	T4b	M1 (HEP)	IV	+	1.42596	1.88455333	32.1603224	nuclear

158	F	65	2014	Proximal	Mucinous adenocarcinoma	T3	M1 (HEP)	IV	+	1.21146	1.66283	37.2583494	nuclear
160	F	48	2014	Proximal	Adenocarcinoma	T3	M0	IIIC	+	1.12708	1.18724	5.33768677	=
161	F	48	2012	Proximal	Mucinous adenocarcinoma	T3	M1b(PER9	IVB	+	1.16472	1.15284	-1.0199876	=
162	M	62	2014	Proximal	Mucinous adenocarcinoma	T2	M1b(OTH)	IVB	+	1.16218	1.13358	-2.4608925	=
163	F	66	2014	Distal	Adenocarcinoma	T3	M0	I	-	1.46842	2.17356	48.0203212	>
164	F	85	2016	Proximal	Adenocarcinoma	T1	M0	I	-	1.30061	1.53802667	18.254255	>
165	F	79	2013	Proximal	Adenocarcinoma	T3	M0	IIA	-	0	1.93532		nuclear
166	F	68	2014	Proximal	Adenocarcinoma	T2	M0	I	-	1.48649	1.53318	3.14095621	=
167	F	73	2013		Adenocarcinoma	T2	M0	I	-	1.26766	1.50488667	18.7137455	>
168	M	84	2015	Proximal	Adenocarcinoma	T1	M0	I	-	1.38756	1.48408667	6.956576	=
169	M	85	2015		Adenocarcinoma	T2	M0	I	-	1.28657	1.89281	47.1206386	>
170	F	94	2014	Proximal	Adenocarcinoma	T4b	M0	IIC	-	1.5016	2.07381	38.1066862	>
171	F	75	2013	Proximal	Adenocarcinoma	T3	M0	IIB	-	1.19706	1.38278	15.5146776	>
172	F	70	2014	Proximal	Adenocarcinoma	T3	M0	IIIB	-	1.27642	1.3536	6.04659908	=
173	M	75	2014	Proximal	Mucinous adenocarcinoma	T4b	M1A	IVA	-	1.21124	1.21867	0.61342096	=
174	F	44	2016	Proximal	Adenocarcinoma	T3	M1b	IVB	-	1.17648	1.69005333	43.6533842	nuclear
175	F	75	2016	Proximal	Adenocarcinoma	T3	M1b(HEP;LYM)	IVB	-	1.24702	1.48963	19.4551812	>
177	M	29	2008	Distal	Adenocarcinoma	T3	M0	II	-	1.24832	1.83452	46.959113	>
178	F	40	2012	Distal	Adenocarcinoma	T3	M0	II	-	1.17729	1.22967	4.44920113	nuclear
179	M	71	2016	Proximal	Adenocarcinoma	T3	M0	II	-	1.19968	1.3417	11.8381568	nuclear
180	M	47	2013	Distal	Adenocarcinoma	T3	M1(HEP)	IV	-	1.7721	2.08488	17.6502455	>
181	F	76	2015	Proximal	Adenocarcinoma	T3	M1(HEP)	IV	-	1.40261	2.38319	69.9110943	>
182	F	82	2012	Proximal	Adenocarcinoma	T4b	M1A	IVA	-	1.52619	1.70444	11.6794108	>
185	M	41	2015	Proximal	Adenocarcinoma	T4b	M0	II	-	1.66496	1.9941	19.7686431	>
186	F	65	2016	Proximal	Mucinous adenocarcinoma	T3	M0	II	-	1.68411	1.791585	6.38170903	nuclear
187	F	77	2016	Proximal	Adenocarcinoma	T4a	M0	IVA	-	1.18561	1.85065333	56.0929255	nuclear

[1] M = male; F = female;

[2] HEP = liver; PUL = lung; PER = peritoneal; OSS = bones; LYM = distant lymph nodes; OTH = other

Table S2.

Comparison of CK2α expression in 143 MLH1-proficient CRCs

	Total (n = 143; 100%)	high nuclear/cytoplasmic (n = 76; 53.1%)	high nuclear (n = 22; 15.4%)	low nuclear/cytoplasmic (n = 45; 31.5%)
**Sex category**				
Female	68 (47.55%)	33 (43.42%)	13 (59.1%)	22 (48.89%)
Male	75 (52.45%)	43 (56.57%)	9 (40.9%)	23 (51.11%)
**Age at diagnosis (SEM)**	69.03 (1.08)	71.5 (1.26)	66.41 (2.94)	66.16 (2.23)
**Localization**				
Distal	56 (39.16%)	33 (43.42%)	6 (27.27%)	17 (37.78%)
Proximal	82 (57.34%)	40 (52.63%)	15 (68.19%)	27 (60.00%)
Unknown	5 (3.50%)	3 (3.90%)	1 (4.55%)	1 (2.22%)
**Year of diagnosis and operation**				
2010	1 (0.70%)			1 (2.22%)
2011	10 (6.99%)	3 (3.95)	2 (9.09%)	5 (11.11%)
2012	29 (20.28%)	15 (19.74%)	4 (18.18%)	10 (22. 22%)
2013	33 (23.07%)	15 (19.74%)	5 (27.73%)	13 (28.89%)
2014	28 (19.58%)	16 (21.05%)	4 (18.18%)	8 (17.78%)
2015	19 (13.29%)	12 (15.79%)	2 (9.09%)	5 (11.11%)
2016	23 (16.08%)	15 (19.74%)	5 (27.73%)	3 (6.67%)
**Tumor**				
pT1/pT1a	10 (6.99%)	3 (3.95%)		7 (15.56%)
pT2	28 (19.58%)	20 (26.32%)		8 (17.78%)
pT3	85 (59.44%)	43 (56.58%)	19 (86.36%)	23 (51.11%)
pT4/pT4a/pT4b	20 (13.97%)	10 (13.16%)	3 (13.64%)	7 (15.56%)
**Metastases**				
M0	104 (72.73%)	57 (75%)	13 (59.09%)	34 (75.56%)
M1	39 (27.27%)	19 (25%)	9 (40.91%)	11 (24.44%)
**UICC-Stage**				
I	30 (20.98%)	19 (25%)		11 (24.44%)
II	41 (28.67%)	21 (27.63%)	5 (22.73%)	15 (33.33%)
III/IIIA/IIIB/IIIC	40 (27.97%)	22 (28.95%)	8 (36.36%)	10 (22.22%)
IV/IVA/IVB	33 (23.08%)	14 (18.42%)	9 (40.91%)	8 (17.78%)
**Histology**				
Adenocarcinoma	131 (91.61%)	71 (93.42%)	21 (95.45%)	39 (86.67%)
Mucinous A.[1]	11 (7.69%)	4 (5.26%)	1 (4.55%)	4 (8.89%)
Mucinous producing A. [2]	2 (1.40%)			2 (4.44%)
Neuroendocrine	1 (0.70%)	1 (1.32%)		

[1] Mucinous Adenocarcinoma
[1] Mucinous producing Adenocarcinoma

Table S3.

Comparison of CK2α expression in 22 MLH1-deficient CRCs

	Total (n = 22; 100%)	high nuclear/cytoplasmic (n = 12; 54.5%)	high nuclear (n = 6; 27.3%)	low nuclear/cytoplasmic (n = 4; 18.2%)
**Sex category**				
Female	15 (68.12%)	8 (66.67%)	5 (83.33%)	2 (50.00%)
Male	7 (31.82%)	4 (33.33%)	1 (16.67%)	2 (50.00%)
**Age at diagnosis (SEM)**	68.23 (3.67)	69 (5.72)	62.67 (6.85)	74.25 (3.57)
**Localization**				
Distal	16 (72.73%)	7 (58.33%)	5 (83.33%)	4 (100.00%)
Proximal	4 (18.18%)	3 (25.00%)	1 (16.67%)	
Unknown	2 (9.09%)	2 (16.67%)		
**Year of diagnosis and operation**				
2008	1 (4.55%)	1 (8.33%)		
2012	2 (9.09%)	1 (8.33%)	1 (16.67%)	
2013	4 (18.18%)	3 (25.00%)	1 (16.67%)	
2014	5 (22.73%)	2 (16.67%)		3 (75.00%)
2015	4 (18.18%)	3 (25.00%)		1 (25.00%)
2016	6 (27.27%)	2 (16.67%)	4 (66.67%)	
**Tumor**				
pT1/pT1a	2 (9.09%)	1 (8.33%)		1 (25.00%)
pT2	3 (13.64%)	2 (16.67%)		1 (25.00%)
pT3	12 (54.55%)	6 (50.00%)	5 (83.33%)	1 (25.00%)
pT4/pT4a/pT4b	5 (22.73%)	3 (25.00%)	1 (16.67%)	1 (25.00%)
**Metastases**				
M0	16 (72.73%)	8 (66.67%)	5 (83.33%)	3 (75.00%)
M1	6 (27.27%)	4 (33.33%)	1 (16.67%)	1 (25.00%)
**UICC-Stage**				
I	6 (27.27%)	4 (33.33%)		2 (50.00%)
II	8 (36.36%)	4 (33.33%)	4 (66.67%)	
III/IIIA/IIIB/IIIC	1 (4.55%)			1 (25.00%)
IV/IVA/IVB	7 (31.82%)	4 (33.33%)	2 (33.33%)	1 (25.00%)
**Histology**				
Adenocarcinoma	20 (90.91%)	12 (100.00%)	5 (83.33%)	3 (75.00%)
Mucinous A. [1]	2 (9.09%)		1 (16.67%)	1 (25.00%)

[1] Mucinous Adenocarcinoma

Table S4.

Comparison of CK2α expression in an exploratory cohort of 23 MLH1-proficient CRCs

	**Total** (n = 23; 100%)	**high nuclear/cytoplasmic** (n = 11; 47.8%)	**high nuclear** (n = 8; 34.8%))	**low nuclear/cytoplasmic** (n = 4; 17.4%)
**Sex category**				
Female	11 (47.83%)	5 (45.45%)	2 (50.00%)	4 (50.00%)
Male	12 (52.17%)	6 (54.55%)	2 (50.00%)	4 (50.00%)
**Age at diagnosis (SEM)**	69.7 (2.55)	69.9 (3.29)	81.75 (4.27)	67.75 (2.6)
**Localization**				
Distal	6 (23.09%)	5 (45.45%)		1 (12.50%)
Proximal	16 (69.57%)	6 (54.55%)	3 (75.00%)	7 (87.50%)
Unknown	1 (4.35%)		1 (25.00%)	
**Year of diagnosis and operation**				
2011	2 (8.70%)		1 (25.00%)	1 (12.50%)
2012	8 (34.78%)	5 (45.45%)	2 (75.00%)	1 (12.50%)
2013	3 (13.04%)	1 (9.10%)	1 (25.00%)	1 (12.50%)
2014	4 (17.39%)	1 (9.10%)		3 (37.50%)
2015	2 (8.70%)	1 (9.10%)		1 (12.50%)
2016	4 (17.39%)	3 (27.27%)		1 (12.50%)
**Tumor**				
pT1/pT1a	1 (4.35%)			1 (12.50%)
pT2	5 (21.74%)	3 (27.27%)		2 (25.00%)
pT3	14 (60.87%)	6 (54.55%)	3 (75.00%)	5 (62.50%)
pT4/pT4a/pT4b	3 (13.04%)	2 (18.18%)	1 (25.00%)	
**Metastases**				
M0	18 (78.26%)	9 (81.82%)	3 (75.00%)	6 (75.00%)
M1	5 (21.74%)	2 (18.18%)	1 (25.00%)	2 (25.00%)
**UICC-Stage**				
I	5 (21.74%)	3 (27.27%)		2 (25.00%)
II	7 (30.43%)	3 (27.27%)	1 (25.00%)	3 (37.50%)
III/IIIA/IIIB/IIIC	7 (30.43%)	3 (27.27%)	2 (50.00%)	2 (25.00%)
IV/IVA/IVB	4 (17.39%)	2 (18.18%)	1 (25.00%)	1 (12.50%)
**Histology**				
Adenocarcinoma	21 (91.30%)	11 (100.00%)	4 (100.00%)	6 (75.00%)
Mucinous A.[1]	2 (8.70%)			2 (25.00%)

[1] Mucinous Adenocarcinoma

Figure S1

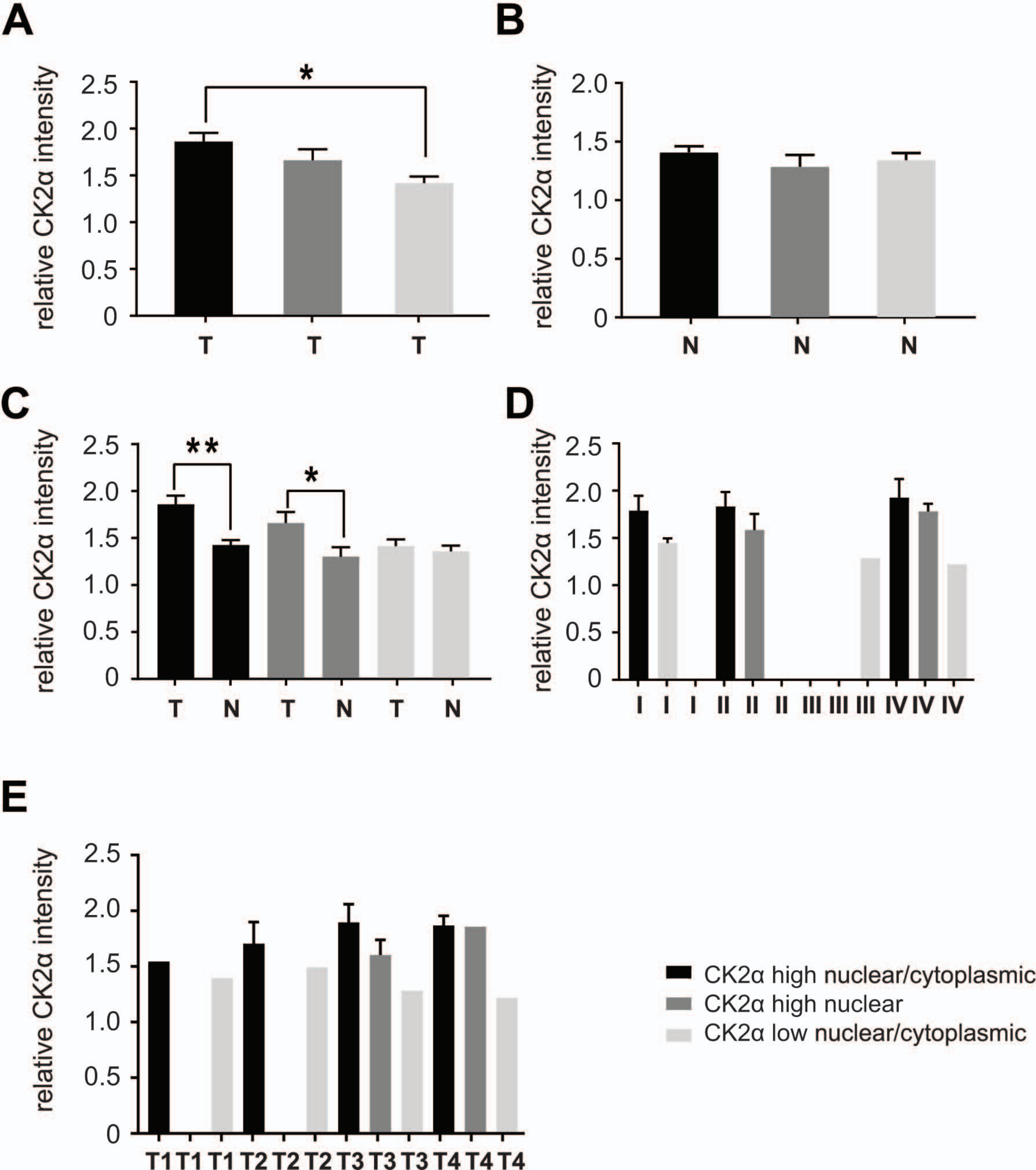

A
B
C
D
E
relative CK2α intensity
2.5
2.0
1.5
1.0
0.5
0
*
**
T T T
N N N
T N T N T N
I I I II II II III III III IV IV IV
T1 T1 T1 T2 T2 T2 T3 T3 T3 T4 T4 T4
CK2α high nuclear/cytoplasmic
CK2α high nuclear
CK2α low nuclear/cytoplasmic

Figure S2

Somatic SNPs identified in the CK2α promoter region of CRCs

3121 acttcacagc tacttatctt ttcctttcgt **agcacttatt gctacctgaa attactttgt**

-1850

g.3227G>C

3181 **ttatgcattt gtttacctgt gcattacttg cttctcactt tagaaggtaa gctccaccag**

3241 **tgagcaactg gtgtatgtct tcctcataac acatccccag tacctagcac agtgtctggc**

3301 **tcatagcaaa tgatgaattg ttgtgagtta aacaaatgtg aatacagttg ggaaaaacaa**

g.3396G>R g.3405G>A; g.3406T>C g.3418G>A

3361 **agccacaaga gccaaaaaga tacgttcgag aggtaggtag aagggtatca tttcctggaa**

g.3424G>A; g.3425G>A g.3430C>T

3421 **gatggcttcc cagaaacttt gggattatct tgtataacct cctcagtcca caagtgggga**

g.3501A>G g.3510G>A g.3524G>A g.3528C>T g.3533G>A

3481 **aattgaggcc cagttacagg acactgagag aaaaactgtt gtgggttccc aggcaattgg**

g.3551C>T g.3557C>Y g.3561C>Y g.3563A>G g.3585C>T g.3591C>T

3541 **gatcagatgt cacaagctgt ctagcagatg aggtccagag aaggccacag ccacctccct**

g.3625C>T g.3631C>T

3601 **gaggccatca ctataagtct gcagccacca ccagccctaa ccccacatgg ggaagaggtg**

g.3661C>T

3661 **cctctttggg agcagaggtg ctagtcagct gacaaactgt gaaggcaggg ctgtggaggg**

3721 **tggagaggcc cattcctgag acaaccagct attctgagac agttagggat aatggttggt**

3781 **actgaaagaa cctggcctcc tcaccctcgt tccagtgctc acacctctgt cccaccagag**

3841 **gtgagactaa tacttgaact cctttcagca actcttagac gcttcagctt ttatcagggc**

g.3944G>T; g.3945G>A

3901 **ctgcaaagga gaggttgatc atcagatgga gccctgtcca ctggggctc ccagtctgat**

g.4018G>A

3961 **ggaggagaca gactgaggtc ctcaggttac tgactgatag tgtctgggtt ggagggagtt**

g.4078G>A

4021 **aaacagagag gagaccccta gaggaaagga tccctgaatt agttcaatcg gcatacagaa**

g.4091C>G

4081 **attatccagt ccatgaggtg acagtaagag aagtaactgc cagtgtaaag ggagggaggc**

4141 **aacacagcta agttccagga attgtttgca gtcttgtggg tggaatcttc cggaatttga**

g.4231G>A

4201 gaagagagtg gcagaatagg aggctggaa ggagggtttg gccaaattat ggaggactct

g.4315G>A

4261 gaatgtcaag tgaagagttt gggctatctt tggtgcataa agaggtccat gggagatttt

g.4333G>A g.4343A>G g.4371G>A

4321 tgaccatagg gagaccatga tgagctctgc atggtagaaa gtgctttgtg gctgttttgg

g.4423G>C

4381 ggaggtagct cgggcaagga gagcaggggt gggaggcaga tagaaggaaa aaaaaacact

g.4449G>A g.4453C>T g.4463G>A g.4498G>A

4441 gtattttgga cgcacagctc ttggcaggac tcctgtagga ggaaggaatt gggccttggt

4501 ctcttgagag ctagtatcct cacttctcag actcctgcgc catgcccttc ctagggtcca

4561 actttcattc acttcttccg aactccaagt ttgaatagaa agtgttgctg aaaccctaat

4621 ttaaaacgag ggtcagagc aaaagagact tcagctccca gaatgcttgg ctctacagct

4681 ctgcaggtcg cgcatggttc ttttcaagaa agggggcca gctgggtgaa gtgtgggaaa

g.4743T>A
g.4742C>T g.4751G>A g.4792A>G

4741 cctgggtacc gccatcttaa cttgggtcaa accaactgtt cacctaatgg gaggttcgtg

g.4805C>T g.4814C>T g.4819G>A g.4842C>T

4801 ttctctctgt tgtcacttgt taacactatt gaaataaaat gcgcttaact attgcaagtc

g.4892C>T

4861 tccttattct gctttgcgtt ggggattcct tccattttgc accctaggcc agaactgaat

g.4939A>G g.4966G>T

4921 ccctaaggtt acaataggac atcccagcat ggccgcattc agagagattc ctctgggggc

g.5018G>A
g.4983A>T g.5016G>T g.5032G>T

4981 ggagtcggaa gctgtctcgc Cccgcctcct ggtaggaggg ggtttccgct tccggcagca

-1

Start of transcription

g.5046T>G g.5073G>A g.5084C>T

5041 gcggctgcag cctcgctctg gtccctgcgg ctggcggccg agccgtgtgt ctcctcctcc

g.5154C>T

5101 atcgccgcca tattgtctgt gtgagcagag gggagagcgg ccgccgccgc tgccgcttcc

5161 accacaggta cctagggagc agccaggcga ggtcgttggc gggtggggtg ggcccaggat

5221 agggcagcgg agcgcggccg accctcacgc ttccagcaga ctcctgagcg gcccgtcgcc

5281 ttcccctgcc cccaccttca cacataaacc aagcgaggcc cctgcagccc atcggggagg

5341 cccaggggcc acccctcagg gcggagggcg aggtttgggg gcagaggccg atgtgagggt

+364

Supplementary Figure S2. Somatic SNPs identified in the CK2α promoter region of CRCs. CK2α is differential expressed in CRCs of patients. In order to analyze underlying molecular changes, the CK2α promotor region -1850 to +364 described by *Winkner et al*. [26] was examined by sequencing. Genomically assigned nucleotides (based on NG_019970.2) in red letters have been identified as variants in the current analysis. Nucleotide marked in black have been identified previously by database search (https://www.ncbi.nlm.nih.gov/snp/?term=CSNK2A1&cmd=DetailsSearch. Numbers on the left refer to genomic CK2α reference sequence (NC_000020.11:472498-543790).
